# A Novel Mutation Causing Pseudohypoparathyroidism 1A with Congenital Hypothyroidism and Osteoma Cutis

**DOI:** 10.4274/jcrpe.v1i5.244

**Published:** 2009-08-06

**Authors:** Tamar Lubell, Maria Garzon, Kwame Anyane−Yeboa, Bina Shah

**Affiliations:** 1 Department of Pediatrics, New York University Medical Center, New York, USA; 2 Department of Dermatology, Columbia University, New York, USA; 3 Department of Pediatrics, Columbia University, New York, USA; 4 Department of Pediatric Endocrinology, New York University Medical Center, New York, USA; +90 804−747−5087+90 804−828−3256bina.shah@nyumc.orgDepartment of Pediatrics, New York University Medical Center, 550 First Avenue, New York, NY 10016

**Keywords:** Pseudohypoparathyroidism, osteoma cutis, congenital hypothyroidism

## Abstract

Various inactivating mutations in guanine nucleotide−binding protein, alpha−stimulating activity polypeptide1 (GNAS1) gene have been described with poor phenotype correlation. Pseudohypoparathyroidism type 1a (PHP1a) results from an inactivating mutation in the GNAS1 gene. Hormone resistance occurs not only to parathyroid hormone (PTH), but typically also to other hormones which signal via G protein coupled receptors including thyroid stimulating hormone (TSH), gonadotropins, and growth hormone releasing hormone. In addition, the phenotype of Albright hereditary osteodystrophy (AHO) is observed, which may include short stature, round facies, brachydactyly, obesity, ectopic soft tissue or dermal ossification (osteoma cutis) and psychomotor retardation with variable expression.

We present a 2−year−old boy with PHP 1A who initially presented at age 3 weeks with congenital hypothyroidism. By 17 months of age, he manifested osteoma cutis, psychomotor retardation, obesity, brachydactyly and resistance to PTH with normocalcemia and mild hyperphosphatemia.

Genetic analysis revealed a novel mutation in exon 13 of GNAS1 in our patient. This mutation, c.1100_1101insA, resulted in a frameshift and premature truncation of bases downstream. This mutation was also found in the mother of this patient who was also noted to have short stature, obesity, brachydactyly and non progressive osteoma cutis, but no hormone resistance.

We report a novel heterozygous mutation causing PHP1A with PTH and TSH resistance and AHO which has not been described previously. PHP1A is also a rare presentation of congenital hypothyroidism.

**Conflict of interest:**None declared.

## INTRODUCTION

Pseudohypoparathyroidism (PHP) represents a heterogeneous group of disorders characterized by end−organ unresponsiveness to PTH leading to hypocalcemia, hyperphosphatemia, and elevated PTH concentrations. PHP is divided into types 1a, 1b, 1c and type 2 that differ with respect to phenotype and hormone resistance patterns. PHP type 1 is associated with a diminished urinary cAMP and phophaturic response to exogenous PTH administration (1). In addition to hormone resistance, patients with PHP type 1a also display a constellation of findings known as Albright hereditary osteodystrophy (AHO). AHO may be characterized by short stature, round facies, brachydactyly, obesity, ectopic soft tissue or dermal ossification (osteoma cutis), developmental delay and reduced Gsa activity (2). The features of AHO are also present in pseudopseudohypoparathyroidism (PPHP), but PPHP differs in that there is no associated hormone resistance. PHP 1b is characterized by PTH−resistance with hypocalcemia and hyperphosphatemia but without the features of AHO. Patients with PHP1c differ from PHP1a patients in that they have multiple hormone resistance but normal Gsα activity. Finally, patients with PHP2 have a normal cAMP response to PTH but have an impaired phosphaturic response.

We herein report a case of PHP1a presenting with congenital hypothyroidism, PHP, osteoma cutis, obesity and psychomotor retardation. These manifestations of PHP1a resulted from a novel mutation in the guanine nucleotide−binding protein, alpha−stimulating activity polypeptide 1 (GNAS1) gene.

## CASE REPORTS

The proband was born full term via cesarean section. His birth weight was 3458 g and length was 50cm. His mother had an uncomplicated pregnancy. He was found to be jaundiced on day three of life with resolution of his hyperbilirubinemia after 24 hours of phototherapy. Neonatal thyroid function screening revealed a plasma total thyroxine of 8.6 ug/dL (normal range 10 to 20  ug/dl) and an elevated thyroid stimulating hormone (TSH) of 32 uU/mL (normal range <20 uU/mL) on post natal day 2, compatible with congenital hypothyroidism.

On initial examination at three weeks of age, the patient’s weight was 3855 g. Physical findings, with the exception of an umbilical hernia, were unremarkable. The anterior fontanel was open and the posterior fontanel was closed. There was no goiter. There was no hypotonia. He had normal prepubertal male genitalia with descended testes bilaterally. The patient was started on Levothyroxine 25 micrograms, at 3 weeks of age, at which time his free T4 was 1.0 ng/dl (normal range 0.8 to 2.2 ug/dl) and his TSH was 12.8 uU/mL (normal range 0.5 to 4.5 uU/mL). A sonogram of the neck was performed at 5 weeks of age which showed a right thyroid lobe measuring approximately 15 x 8 x 8 mm (right thyroid lobe volume of 0.5 cc). There was a solitary nodule in the mid aspect of the right lobe of the thyroid measuring 5.0 x 3.0 x 4.0 mm. The left lobe of the thyroid measured 12 x 7 x 7 mm, (left thyroid lobe volume of 0.3cc) with no nodules identified. A repeat sonography at 8 months of age showed a stable appearance of the right lobe nodule. 

At 17 months of age, subcutaneous ossification was noted on the left thumb. Subsequent radiologic examination of the hands showed an area of calcification at the left first metacarpal−phalangeal joint as well as diffuse osteopenia,  and an advanced bone age of 4 years at a chronologic age of 26 months. Serum biochemistry  at 21, 25 and 28 months of age revealed PTH levels of  98, 228 and 237 pg/mL respectively (normal range 15−75 pg/ml), marginally elevated phosphate levels (6.7, 6.5 and 6.3 mg/dL, normal range 4.5−5.5 mg/dL) and calcium levels of 10.1, 9.5 and 9.3 mg/dL (normal range 8.3−10.3 mg/dl). The 25−hydroxy vitamin D level was 27 ng/ml (normal range 30−80 ng/ml) and 1,25−dihydroxy vitamin D level was 64 pg/ml (normal range 15−75 pg/ml) at 27 months. 

A review of the patient’s growth data revealed that his height corresponded to 81^st^ centile for age (Z score 0.864) at 13 months of age and to 76^th^ percentile (Z score 0.706) at 25 months of age. His weight centiles changed from 86^th^ centile (Z score 1.084) to over 99^th^ centile (Z score 2.377) in the same period.  His body mass index at 25 months of age was 20.7 kg/m^2^ (>97^th^ centile, Z score 2.31). 

The patient showed a delayed motor and language development and he is currently receiving occupational therapy, speech therapy and special education. 

The patient’s mother also showed features of AHO including obesity, short 4^th^ and 5^th^ metacarpal bones with multiple areas of ectopic calcium deposits which were present for 21 years and were non progressive. There was no evidence of hormone resistance in the mother; her PTH was 31.5 pg/ml (normal range 10−65 pg/ml). 

**Genetic Study Results**

Mutations in the gene encoding Gsa (GNAS1) were investigated by polymerase chain reaction (PCR) and DNA sequencing of exons 1 (codon 7−43) through 13. A novel mutation, c.1100_1102insA, was detected in exon 13 which resulted in a frameshift and premature termination, 10 amino acids downstream. This mutation was also identified in the patient’s mother. This alteration is not considered as a polymorphism.

## DISCUSSION

PHP has been linked to mutations in the gene encoding the alpha subunit of the stimulatory G protein (GNAS1), which maps to 20q13.2. The inheritance pattern appears to be autosomal dominant and displays variable penetrance. The GNAS1 locus is very complex and gives rise to Gsα and several other splice variants (3, 4). Estimates of the proportion of AHO caused by GNAS1 mutations range from 60 to 90%. These mutations have been described throughout the 13 exon gene. Approximately 35 to 50% of reported mutations are 4 bp deletions in exon 7 and this seems to be an area of increased susceptibility. Mutations in exons 7−10 comprise 35−50% of all mutations (5). 

Gsα is ubiquitously expressed and its deficiency results in resistance to PTH as well as to other hormones that signal through G protein coupled receptors such as TSH and gonadotropins (6, 7). Interestingly, although vasopressin and adrenocorticotropic hormone (ACTH) also signal via G protein coupled receptors, resistance to their actions has not been observed in patients with PHP1a. (7). The haploinsufficiency of the GNAS1 gene is tissue specific and in most tissues transscription occurs equally from both alleles. However, in a subset of tissues such as the proximal renal tubules, anterior pituitary, thyroid gland and ovary, expression is monoallelic and is restricted to the maternal allele. This may help elucidate the selective resistance to hormones observed in patients with PHP (3, 8).  

In our case, we present two individuals (the proband and his mother) with identical GNAS1 mutations who display the characteristic features of AHO, however differ in their resistance to multiple hormones. This finding can be explained by genomic imprinting of Gsα in which maternal transmission leads to offspring with PHP1a whereas paternal transmission leads to offspring with PPHP, as was the case in the patient’s mother. Tissue specific imprinting in addition to "parent oforigin" imprinting explains why Gsα expression is equally reduced in PHP1a and PPHP yet only maternal transmission results in proximal renal tubule resistance to PTH (9).

Congenital hypothyroidism has been reported as the presenting manifestation of PHP (6, 10, 11). TSH levels are typically elevated at birth but may then normalize for a period of months before once again becoming elevated. The resistance to TSH is generally mild, and this may be explained by partial imprinting in the thyroid with incomplete silencing of the paternal allele (8). Goiter is typically absent given that the defect lies in TSH signaling (12) and antithyroid antibodies are also absent (9). A very small thyroid nodule was present since birth in our patient. Although unclear, it may be possible that this thyroid nodule could be linked to TSH resistance.

The terms calcinosis cutis and osteoma cutis are often used interchangeably in the literature when describing the subcutaneous manifestation of PHP1a. Calcinosis cutis differs from osteoma cutis in that it describes a group of disorders in which insoluble compounds of calcium are deposited within the skin rather than true bone (as is the case in osteoma cutis). Ectopic bone formation (osteoma cutis and progressive osseous heteroplasia) has been observed in patients with AHO. Progressive osseous heteroplasia (POH) is a form of heterotopic ossification (HO) that progresses from superficial tissues into the deeper connective tissues including muscle and fascia but is not associated with AHO or hormone resistance. It is caused by paternal inheritance of the GNAS1 mutation (13). These syndromes are all associated with heterozygous mutations in GNAS1 which leads to decreased expression or function of Gsα.  This decreased Gsα activity has been directly linked to osteogenic differentiation in human mesenchymal cells and this finding provides a potential explanation for the ectopic bone formation in these syndromes (14). This results in a spectrum of extra−skeletal ossification disorders with POH and progressive heterotopic ossification syndromes lying at the far end of the phenotypic spectrum. Although PHP1a is typically associated with a non progressive form of heterotopic ossification, PHP1a with a progressive form of HO has been described (15). There is no specific genotype−phenotype correlation that distinguishes the non progressive forms of heterotopic ossification from the progressive forms (13). 

The pathogenesis of calcinosis cutis is not completely understood and it may develop in response to local and/or systemic factors such as collagen vascular diseases, cutaneous neoplasms, infections and trauma (16). Given that calcinosis cutis is not found in patients with primary hypoparathyroidism, it is less likely that hypocalcemia and hyperphosphatemia are contributing to this clinical picture (17). Moreover, in case reports describing calcinosis cutis in patients with PHP1a, the calcium levels were normal or only marginally above reference values (17, 18). Intracranial calcifications and cataracts have also been observed in patients with hypoparathyroidism and PHP. Unlike calcinosis cutis, these calcifications do appear to be caused by the hypocalcemia and hyperphosphatemia (1). It is unclear if calcinosis cutis lies at the milder end of the spectrum of heterotopic ossification. Besides osteoma cutis, other features of AHO may include obesity, psychomotor retardation and tall stature with advanced bone age. Studies have implicated haploinsufficiency of Gsα as a cause of accelerated differentiation of chondrocytes and osteoblasts and premature epiphyseal fusion leading to an advanced bone age (8). 

The mainstay of treatment for PHP involves the administration of oral calcium and 1 α−hydroxylated vitamin D metabolites, such as calcitriol. This therapy should be initiated in every patient with a diagnosis of PHP with the goal of maintaining serum total and ionized calcium levels within the reference range to avoid hypercalciuria and to maintain PTH levels in the normal range.PTH indirectly activates osteoclasts which are involved in bone resorption and accelerates bone remodeling. In patients with PHP there is variable responsiveness of the skeleton to PTH despite clinical evidence ofimpaired hormone responsiveness in other tissues including the kidney (19). In the absence of skeletal osteoclast resistance to PTH, hyperparathyroid bone disease such as osteopenia or osteitis fibrosa may develop. Therefore, it is important to maintain PTH in the normal/upper normal range even in the asymptomatic normocalcemic patient (20, 21). Skeletal responsiveness to PTH may help explain the periods of spontaneous normocalcemia observed in some patients (19). The finding of elevated PTH levels in the context of normocalcemia suggests that hypocalcemia may not be the only factor leading to hyperparathyroidism in PHP. Factors contributing to the hyperparathyroidism in PHP may include frequent small unrecognized declines in the serum calcium concentration in addition to a reduction in 1,25−dihydroxy−vitamin D. Other suggested explanations include secretion of an abnormal PTH, or abnormal metabolism of PTH (22). 

In summary, we report a case of PHP 1A presenting with congenital hypothyroidism with a small thyroid nodule and osteoma cutis. The Gs−a mutation can present with variable effects on phenotype and clinical clues provide valuable information.
